# Hydrogel-Based Adsorbent Material for the Effective Removal of Heavy Metals from Wastewater: A Comprehensive Review

**DOI:** 10.3390/gels8050263

**Published:** 2022-04-22

**Authors:** Zenab Darban, Syed Shahabuddin, Rama Gaur, Irfan Ahmad, Nanthini Sridewi

**Affiliations:** 1 Department of Chemistry, School of Technology, Pandit Deendayal Energy University, Raisan 382426, India; darbanzenab05@gmail.com; 2 Department of Clinical Laboratory Sciences, College of Applied Medical Sciences, King Khalid University, Abha 61421, Saudi Arabia; irfancsmmu@gmail.com; 3 Department of Maritime Science and Technology, Faculty of Defence Science and Technology, National Defence University of Malaysia, Kuala Lumpur 57000, Malaysia

**Keywords:** hydrogels, heavy metals removal, wastewater

## Abstract

Water is a vital resource that is required for social and economic development. A rapid increase in industrialization and numerous anthropogenic activities have resulted in severe water contamination. In particular, the contamination caused by heavy metal discharge has a negative impact on human health and the aquatic environment due to the non-biodegradability, toxicity, and carcinogenic effects of heavy metals. Thus, there is an immediate need to recycle wastewater before releasing heavy metals into water bodies. Hydrogels, as potent adsorbent materials, are a good contenders for treating toxic heavy metals in wastewater. Hydrogels are a soft matter formed via the cross-linking of natural or synthetic polymers to develop a three-dimensional mesh structure. The inherent properties of hydrogels, such as biodegradability, swell-ability, and functionalization, have made them superior applications for heavy metal removal. In this review, we have emphasized the recent development in the synthesis of hydrogel-based adsorbent materials. The review starts with a discussion on the methods used for recycling wastewater. The discussion then shifts to properties, classification based on various criteria, and surface functionality. In addition, the synthesis and adsorption mechanisms are explained in detail with the understanding of the regeneration, recovery, and reuse of hydrogel-based adsorbent materials. Therefore, the cost-effective, facile, easy to modify and biodegradable hydrogel may provide a long-term solution for heavy metal removal.

## 1. Introduction

### 1.1. Problem Statement

**Figure 1:**
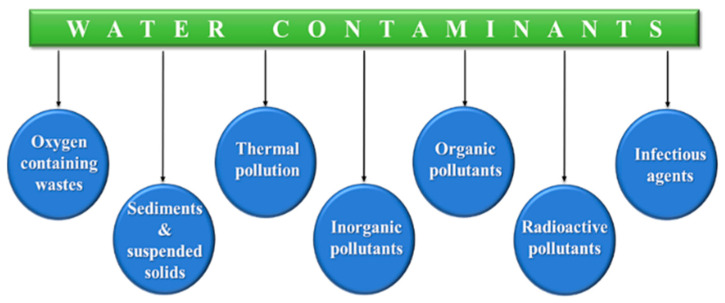
Different pollutants in contaminated water.

Water is essential for all living organisms on the planet. Although, it occupies 71% of the total surface area of the earth, only 3% of water is available as freshwater and less than 1% is potable. The remaining percentage of water is inaccessible in different forms such as ice, glaciers, and snow on the south and north poles [1]. Water plays a significant part in the hydrological cycle, food-processing industries, chemical weathering, domestic usage, agricultural irrigation, and so on. Therefore, there is an increasing need for freshwater, but the availability is limited. Freshwater is contaminated by discarding waste in various water bodies in the form of marine dumping, oil leakage, industrial waste, sewage waste, etc. Different pollutants present in wastewater are summarized in Figure 1. Among them, heavy metals are found to be the most common pollutant in contaminated water, which deteriorates the sustainable environment. Water contamination by heavy metals has harmed human health all around the world due to the fast development in industries, economics, and population [2].

### 1.2. Heavy Metals and Their Hazardous Effect

Heavy metals are referred to as metals with a density of 5 gm/cm^3^ and are poisonous, toxic, and hazardous even at very low concentrations. The sources of heavy metal contamination into water are categorized in two ways: (1) natural ways like soil erosion, rainfall, dissolution of soluble salts, etc., and (2) artificial ways like industrial waste, and urban wastewater [3]. Heavy metals include mercury (Hg), zinc (Zn), arsenic (As), cadmium (Cd), silver (Ag), iron (Fe), lead (Pb), tin (Sn), and the platinum group of metals. Heavy metals are non-biodegradable elements [4] that cause detrimental effects on the natural ecosystem and human health when their concentration goes beyond permissible limits. For instance, persistent intake of inorganic arsenic causes lung, bladder, skin, and kidney cancer in humans via consumption of drinking water [5]. Mercury accumulation in the food chain shows a negative impact on human health such as kidney and pulmonary function impairment, chest pain, and damage to the central nervous system [6]. Some other examples are listed in Table 1.

Heavy metals are not degraded by natural mechanisms and hence persist in the environment for a long duration of time. They may be converted into insoluble compounds or other forms. Water, air, and soil are the three key environmental compartments that get affected by heavy metal contamination (Table 1). Runoffs from cities, villages, towns, and factories transport the heavy metals that accumulate in a flowing stream. Even if a low concentration is transferred to water streams, it is extremely harmful to humans and the natural ecosystem [7]. Air pollution is caused by dust and particulate matters such as PM_2.5_ and PM_10_ which are discharged by various natural and anthropogenic processes. Soil erosion, dust storms, rock weathering, and volcanic eruptions are examples of natural processes that release particulate matter in the air, whereas anthropogenic activities are mainly transport-related and industrial. These particulate matters cause corrosion, haze, and eutropication, and lead to the formation of acid rains [8]. Heavy metals pollute soil by damping wastes like animal manures, pesticides, fertilizers, sewage sludge, spillage of petroleum distillates, etc. The use of this untreated waste has resulted in a high concentration of heavy metals in agricultural fields, which affects the entire biosphere. They are directly absorbed by plants, causing a risk to the plant and the food chain that consumes it. They affect soil qualities such as color, pH, and porosity and also pollute water [9]. Therefore, it is urgent and necessary to remove toxic heavy metals from contaminated wastewater. A variety of wastewater recycling techniques have been developed, which are further discussed in this review article.

**Table 1:** Toxic effects of different heavy metals on human health [10,11].

Heavy Metals	Leading Source	Path of Entry	Toxic Effects on Human Health	Environmental Hazards	MCL (mg/L)
Lead (Pb)	Mining, automobile emissions, smoking, pesticide, paint, burning of coal	Ingestion and inhalation	Damages the central nervous system, fetal brain, kidney, reproductive system, liver, basic cellular processes, and causes diseases, namely, anemia, nephrite syndrome, hepatitis, etc.	Soil and water pollution	0.015
Cadmium (Cd)	Pesticide fertilizer, electroplating, Cd-Ni batteries, welding	Ingestion and inhalation	Irritation of respiratory system, damages liver, kidney, and lungs	Soil and water pollution	0.005
Nickel (Ni)	Electrochemical industries	Inhalation	Causes lung, kidney, and gastronomical pain, renal edema, pulmonary fibrosis, and skin dermatitis	Soil and water pollution	0.1
Zinc (Zn)	Plumbing, refineries, metal plating, brass manufacture	Ingestion, inhalation, and through skin	Vomiting, pain in the stomach, skin irritation, nausea, and anemia	Soil and water pollution	0.8

## 2. Methods Used for Recycling Wastewater

**Figure 2:**
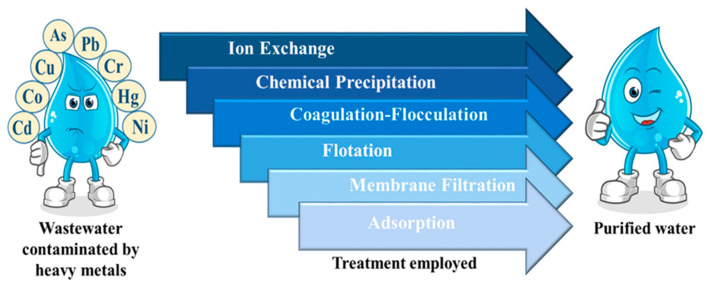
Wastewater recycling methods.

Over the years, numerous methods have been developed to remediate heavy metal-contaminated wastewater before discharging it into the environment. Heavy metals can be removed from wastewater by using a variety of methods, including ion exchange, coagulation-flocculation, flotation, membrane filtration, chemical precipitation, and adsorption (Figure 2). However, each method has its own set of advantages and disadvantages (Summarized in Table 2).

Among the presented methods in Figure 2, adsorption is considered to be one of the most efficient, low-cost, and simple-to-operate methods to remove heavy metals from contaminated water when compared with other methods [12]. Moreover, the adsorption process is technologically feasible and attractive, as the adsorbent material can be reused and regenerated. In this process, no secondary waste is generated during the removal of heavy metals [13]. The process also has the advantage of removing low concentrations of heavy metals from the solution with low energy consumption [14,15]. Adsorption is a mass transfer surface phenomenon that leads to the binding of molecules from liquid bulk (adsorbate) onto the solid surface (adsorbent) [16]. This binding occurs due to the presence of residual imbalance forces that attracts and retains molecules on the surface of the solid or liquid phase [17]. The adsorbent adsorbs the adsorbate via bonding interactions like a covalent bond or Van der Waals forces [18].

Over the years, researchers have developed adsorbent materials for the removal of toxic heavy metals from wastewater like rice husk bio-char, sugar beet pulp, TiO_2_, activated carbon, clay, etc. [19,20,21,22,23]. However, these adsorbent materials suffer from certain disadvantages such as their difficulty separating from the water after the decontamination process, higher production cost, economic unsustainability for large-scale applications, and many other reasons [24]. This calls for the immediate development of an adsorbent material that is cost-effective, easy to handle, biodegradable, and biocompatible adsorbent material to purify contaminated water. In recent years, hydrogels have gained tremendous attention as the potential adsorbent material owing to their excellent water affinity, controllable swelling behavior, high porosity, better mechanical properties, and easy handling; these are the main factors for the reuse of adsorbent material. Hydrogel-based material has shown substantial attention for applications in different fields, such as biomedicine, agriculture, food additives, drug delivery, wound dressing, regenerative medicine, and cosmetics. Even though hydrogel-based adsorbents have a long history in a few of the above-mentioned fields, their application for contaminant removal from wastewater has been only reported over the last decade. Our emphasis will be on the application of hydrogel-based material for the removal of toxic and hazardous heavy metals from wastewater.

**Table 2:** Advantages and disadvantages of various methods used for recycling wastewater.

Methods	Advantages	Disadvantages	References
Ion exchange	Does not produce a large amount of sludge, easy regeneration of resins	High operational cost, selective towards certain metal ions	[25]
Chemical precipitation	Low capital cost, simple process	Produces a large amount of sludge, ineffective in treating low concentration of heavy metal ions	[26]
Coagulation-flocculation	Easy to employ, inexpensive, low energy consumption	Complete removal of heavy metals is difficult, generation of a large quantity of sludge	[27]
Flotation	Economically efficient	Low elimination efficiency,	[28,29]
Membrane filtration	Small space requirement, high efficiency, high separation selectivity	complex process, high operational expense due to membrane fouling	[30]
Adsorption	Technologically feasible, effective, low-cost adsorbent, no waste generation, easy operation conditions	Low selectivity	[3,13]

## 3. Hydrogels for Removal of Heavy Metals

**Figure 3:**
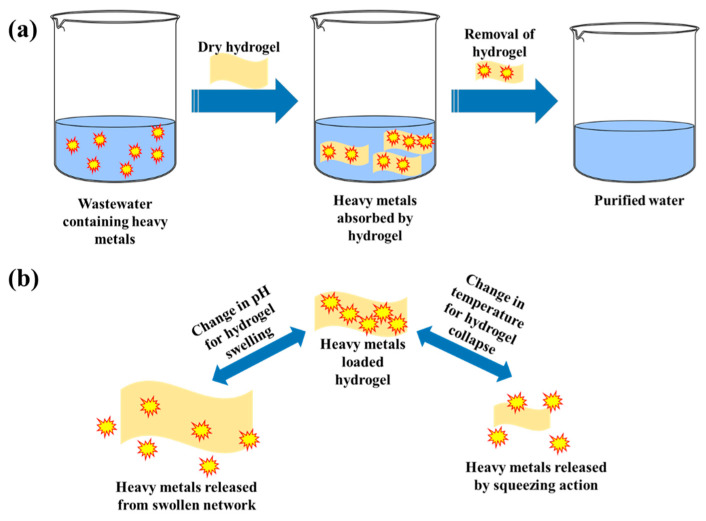
(**a**) Schematic illustration for removal of heavy metals from wastewater by using hydrogel-based adsorbent material. (**b**) Change in structure on applying external stimuli like pH and temperature.

In 1894, the term “hydrogel” was first coined by Bemmelen to explain colloidal gels [31]. DuPont scientists reported the first synthetic hydrogel, poly (2-hydroxyethyl methacrylate) (PHEMA), in 1936 [32]. Witcher and Lim were the first to report the use of PHEMA for the application of contact lenses in 1960 [33]. Since then, hydrogels have been an intriguing topic for researchers, and they now represent a developing and active research field aimed at providing better solutions for various needs in many applicative fields. The use of hydrogels for the extraction of heavy metals from wastewater is becoming more popular, as they can capture and store different heavy metals found in wastewater within their network structure. Hydrogels are regarded as hydrophilic gels, which consists of chemically reactive functional groups and physically distinct three-dimensional (3D) network [34]. The porous three-dimensional network of hydrogel allows absorption and retention of a large volume of water without dissolving [35]. The hydrophilic groups in the polymeric network enable the formation of a flexible structure, which allows easy diffusion of solute into the three-dimensional framework of gels and forms a stable complex with the functional group present on a long polymeric chain [36]. Due to distinct properties like hydrophilicity, biocompatibility, biodegradability, viscoelasticity, and superabsorbancy, hydrogel adsorbents can play a prime role in the capture of heavy metals from contaminated water (Figure 3a) and can discharge these hazardous pollutants upon changes in the external environment (change in pH, temperature, etc.) (Figure 3b) [34,37].

In recent years, hydrogel adsorbents have shown a high potential for the effective elimination of heavy metals. Hydrogel absorbs heavy metals in a three-dimensional interstitial structure ensuring more sites per unit volume [38]. Unlike other adsorbents, hydrogels adsorb heavy metals in a three dimensional, highly porous network that leads to high adsorption efficiency [37]. Adsorption or desorption of heavy metals is mainly due to the surface chemistry and presence of hydrophilic functional groups (−COOH, −NH_2_, −OH, −SO_3_H, etc.) that act as a complexing agent for heavy metal removal from aqueous media [36,37]. Moreover, hydrogels can be modified by the addition of new functional groups or the preparation of composites with natural or synthetic sources to enhance heavy metal absorption capacities [39]. The swelling behavior of hydrogels is associated to an extent with hydrophilic functional groups present in the polymer backbone, the degree of cross-linking, the elasticity of the network, and the porosity of the polymer [40]. The hydrophilic polymers in hydrogel can swell up to several times their original volume in aqueous media and hold large content of water about 400 times its original weight [41]. Hydrogels are insoluble in water which leads to easy regeneration because of the presence of chemically cross-linked polymers, which enhance their mechanical strength as well as decreases the swelling ratio. Therefore, it is necessary to balance the amount of crosslinking and swelling ratio to obtain a stronger hydrogel [42]. Some other advantages of hydrogels are that they can be synthesized with the desired charges, controllable sizes, and functional groups [43]. The three most important parameters on which the capacity of cross-linked hydrogel synthesized depend are: (a) polymer volume fraction of hydrogel in swollen shape, which determines the quantity of fluid absorbed into hydrogel network, (b) the average molecular weight between two cross-links, which determines the degree of cross-linking for the prepared hydrogel, and (c) the network mesh size, which determines the degradability, mechanical strength, and diffusivity of releasing components into hydrogel structure [44].

An ideal hydrogel should have the following characteristics to be widely applied for the effective removal of heavy metals from polluted wastewater [24].

- Cost-effective
- High adsorption capacity to absorb heavy metals from wastewater
- High adsorption rate (determined by porosity and particle size)
- Biodegradable
- Easy to modify
- The low content of the unreacted residual monomer
- High stability and durability during swelling and storage
- Non-toxic, colorless, and odorless
- pH neutrality after swelling in aqueous media
- Deswelling capabilities and re-watering (able to release back the stored water)

## 4. Properties of Hydrogel

An ideal hydrogel has distinctive characteristic properties such as swelling or deswelling in the presence of external stimuli, responsiveness to the change in temperature, pH, light, etc., and biodegradability.

### 4.1. Swelling or Deswelling of Hydrogels

Hydrogels are the cross-linked polymer that swells and imbibes water when immersed in the aqueous media. These three-dimensional structures can swell up to several times their dry weight [45]. Hydrogels can respond to external stimuli (temperature, pH, light, salt, magnetic fields, biomolecules, and ionic strength) by shrinking, swelling, and discoloration [46]. Factors affecting swelling kinetics and equilibrium are the chemical structure of the polymers, cross-linking ratio, synthesis state, and ionic media. Chemical structure affects the swelling of hydrogel; hydrophilic groups swell more in comparison to hydrophobic groups. Cross-linking also has a significant effect on swelling behavior, as a highly cross-linked polymer network will show less swelling and vice versa. The swelling behavior of hydrogels is also affected by temperature and pH [47]. Temperature-sensitive hydrogels undergo swelling or de-swelling (change in volume) with the change in the temperature. These hydrogels swell below the low critical solution temperature and shrink above the low critical solution temperature [48]. pH-sensitive hydrogels undergo swelling or deswelling by varying pH levels. These hydrogels consist of ionizable acidic and basic groups connected to the polymer backbone that can add or release protons by varying pH levels. At the high pH value, the acidic groups on polymer chains deprotonate whereas at low pH values, the basic groups get protonated [49].

Swelling in hydrogels takes place in three steps:—(a) the diffusion of water into the three-dimensional network of hydrogel, (b) loosening of the polymeric chains, and (c) expansion of hydrogel structure. A hydrogel in swollen form is referred to as the rubbery state and the dry form as the glassy state. When dry hydrogel comes in contact with the solvent, the free space in a polymer network permits the solvent to enter the hydrogel matrix easily. This transforms the dry or glassy state into a swollen or rubbery state. The de-swelling of hydrogel occurs when water is removed from a hydrogel matrix [47]. Experimentally, the swelling ratio can be calculated by the following formula [50].

$$\mathrm{Swelling}\;\mathrm{ratio}=\left[\left(W_{s}-W_{d}\right)÷W_{d}\right]\times 100$$

where, *W_s_* and *W_d_* are the weight of the swollen and dry hydrogel, respectively.

### 4.2. Stimuli-Responsive Hydrogels

**Figure 4:**
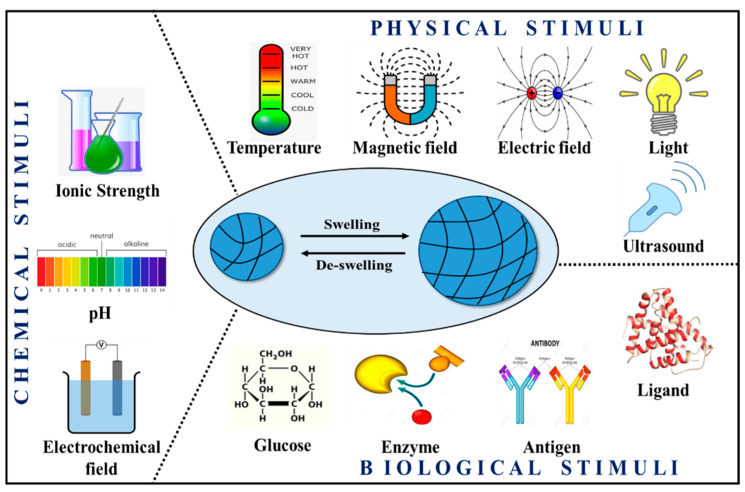
Response of hydrogel to different stimuli.

Gels that respond to changes in the external environment (temperature, pH, magnetic field, electric field, etc.) are termed stimuli-responsive hydrogels. These hydrogels have characteristics to transform their shape (from solution to gel) based on the application [51]. Furthermore, stimuli-responsive hydrogels are categorized into three classes: chemical, physical, and biological. pH, solvency, ionic strength, and electrochemical field are typical chemical stimuli. Physical stimuli include temperature, magnetic field, electric field, light, mechanical force, and ultrasound. Biological stimuli include enzymes, glucose, antigen, ligands, etc. (Figure 4) [52]. Multi-responsive hydrogels are the kind of hydrogels that respond to two or more stimuli [53].

### 4.3. Biodegradable

Biodegradability refers to the ability of the hydrogel to break down into harmless and non-toxic end products by bacteria or other organisms. Hydrogels’ biodegradability is determined by the functional groups present in the system as well as the method of synthesis. The degradation process involves solubilization and hydrolysis of biological entities of hydrogel into safer end products. Biodegradable polymers include a wide range of hydrophilic synthetic and natural polymers. Due to diffusion, these polymers absorb an ample amount of water and expand to a large extent. The breakdown of these polymers is influenced by various parameters such as molecular weight, hydrophilicity, and the interaction of the polymer with water. Other environmental conditions like temperature and pH also influence the breakdown of polymers via solubilization.

Chemical hydrolysis can be used to degrade a variety of polymers that cannot be destroyed by simple hydrolysis. These polymers do not produce hydrogel; rather they mix with hydrogel to form a hydrophilic monomer, which is then combined to form a biodegradable hydrogel. The formed hydrogel undergoes degradation via chemical hydrolysis via ester bonds. Furthermore, hydrogels can be degraded by enzyme hydrolysis and this category of hydrogels involves polymers such as proteins, polysaccharides, and synthetic polypeptides. Enzyme hydrolysis takes place by a set of hydrolases that catalyze the hydrolysis of C-N, C-O, and C-C bonds. Peptidases and proteinases are hydrolases that degrade polypeptide and protein hydrogels, respectively. Moreover, glycosidase is the sole enzyme that degrades polysaccharide hydrogels [47].

## 5. Classification of Hydrogel

**Figure 5:**
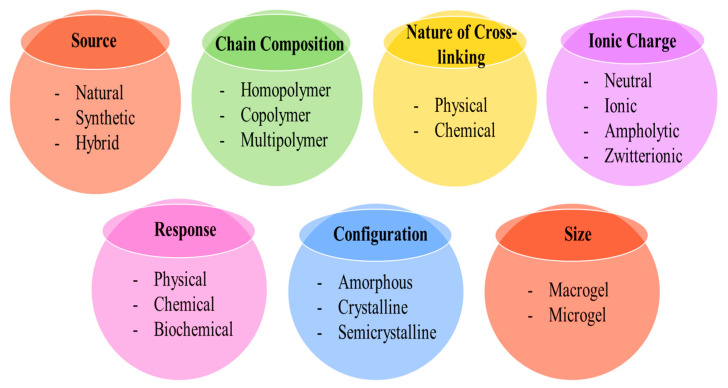
Classification of hydrogels.

Hydrogels are classified depending on their source, nature of cross-linking, chain composition, ionic charge, response to external stimuli, configuration, and size. The most important parameters for the classification of hydrogels are depicted in Figure 5.

### 5.1. Based on Source

Hydrogels can be classified as natural, synthetic, and hybrid.

(a) Natural hydrogels are synthesized by using natural sources including chitosan, agar-agar, cellulose, lignin, gelatin, alginate, dextran, collagen, and many other materials [54].
(b) Synthetic hydrogels are prepared by using synthetic polymers namely hydroxy methyl methacrylate (HEMA), acrylic acid (AA), vinyl acetate (VAc), ethylene glycol (EG), ethyleneglycoldimethacrylate (EGDMA), methacrylic acid, N-vinyl 2-pyrrolidone (NVP), and many other materials [54].
(c) Hybrid hydrogels are made by combining natural and synthetic sources [55]. Zhang et al. prepared chitosan-g-poly (acrylic acid)/attapulgite/sodium humate hydrogel for effective removal of Pb^2+^ [56].

### 5.2. Based on the Nature of Chain Composition

Hydrogels can be classified into three principal classes: homo-polymeric hydrogels, co-polymeric hydrogels, and multipolymer hydrogels.

(a) Homo-polymeric hydrogels are cross-linked polymer-network originating from a single type of monomer unit [57]. The structural unit of these hydrogels depends on the type of monomer, cross-linker, and polymerization technique [58].
(b) Co-polymeric hydrogels are composed of two or more types of monomer units with at least one hydrophilic monomer, arranged in a random, block, and irregular structure along the backbone of the polymer network [59]. These hydrogels are prepared by cross-linking or polymerization between both the monomers by using a cross-linker and initiator. An example of such hydrogels is chitosan, k-carrageenan, carboxymethyl cellulose composite hydrogel which is used to remove metal ions.
(c) Multipolymer hydrogels are cross-linked polymer-network prepared by three or more monomer units via cross-linking and polymerization reactions. For example, Kim et al. synthesized chitosan-based multicomponent functional gel comprising multiwall carbon nanotubes, polyaniline, poly (acrylic acid), and poly (4-amino diphenyl amine) [60].
(d) Interpenetrating polymeric hydrogels are comprised of two independent, intertwined polymer networks, having natural and/or synthetic polymer components. In a semi-interpenetrating polymer hydrogel, one polymer has a linear network that diffuses into another cross-linked network. There is no chemical bonding between the polymers [54].

### 5.3. Based on the Nature of Cross-Linking

Hydrogels can be classified into two categories: physically cross-linked hydrogels and chemically cross-linked hydrogels.

(a) Physically cross-linked hydrogels have a transient junction that arises due to physical interaction such as hydrogen bond, ionic interaction, and hydrophobic interaction.
(b) Chemically cross-linked hydrogels have permanent junctions that arise due to covalent bonds [50].

### 5.4. Based on the Reaction of Hydrogel with the External Stimulus

Hydrogels can be classified into two distinct categories: traditional hydrogels and environmentally sensitive hydrogels.

(a) Traditional hydrogel is not reactive to environmental changes
(b) An environment-sensitive hydrogel can detect changes caused by chemical (pH, concentration), biochemical (antigen, enzyme, ligand), and physical (temperature, pressure, light) factors [50].

### 5.5. Based on the Configuration

On the basis of the physical structure and chemical composition, hydrogels can be classified as amorphous, semi-crystalline (mixture of crystalline and amorphous phases), and crystalline [41].

### 5.6. Based on the Size

Hydrogels can be classified into two classes: macrogel and microgel. The macrogel is further classified as a porous sponge, columnar, membranous, fibrous, and spherical according to its morphology. The prepared microgel also can be classified into nanometer and micron [50].

### 5.7. Based on Ionic Charge

Hydrogels can be classified into four categories based on the electric charge placed on the cross-linked network: neutral, ionic, ampholytic, and zwitterionic.

(a) Neutral hydrogels are also known as non-ionic hydrogels. These hydrogels contain no charge on side groups or polymer backbone.
(b) Ionic hydrogels are further classified as anionic and cationic. Anionic hydrogels carry negatively charged functional groups like sulfonyl, carboxyl, etc. and at high pH values show an increase in swelling behavior. Cationic hydrogels carry positively charged functional groups like amines, thiol, etc., and at low pH values exhibit an increase in swelling behavior.
(c) Ampholytic or amphoteric hydrogels contain acidic as well as basic groups.
(d) Zwitterionic hydrogels contain cationic and anionic groups in their structure [61,62].

## 6. Surface Functional Groups of Hydrogel

A group of atoms in a compound responsible for chemical reactions is known as the functional group. Functional groups play a significant role in determining the chemical reactivity of the molecule as well as the type and strength of intermolecular forces. The paramount functional groups incorporated in a three-dimensional network of hydrogels for metal adsorption are classified into three groups:—(a) nitrogen-containing functional groups, (b) oxygen-containing functional groups, and (c) sulfur-containing functional groups [63]. Table 3 summarizes hydrogels containing different functional groups and removed heavy metals.

### 6.1. Nitrogen-Containing Functional Groups

#### 6.1.1. Amine Group

The amine group contains a nitrogen atom that has a lone pair of electrons, that readily attach to cationic metal ions. The methods used to functionalize the amine group on the hydrogel surface are atom-transfer radical polymerization, formaldehyde treatment, and gamma ray-induced polymerization [63].

#### 6.1.2. Amide Group

The general formula for the amide group is –CONH. In general, amine groups are more often functionalized on the hydrogel surface than amide groups. Moreover, monomers like 2-acrylamido-2-methyl-1-propanesulfonic acid sodium salt have an amide group in the polymer backbone, that complexes with heavy metals Cu(II) and Ni (II) [64].

#### 6.1.3. Quaternary Ammonium Groups

Quaternary ammonium groups [R-N^+^(CH_3_)_3_] show strong attraction toward metal oxyanions (Cr_2_O_7_^2−^, HCrO_4_^−^, AsO_4_^3−^ and CrO_4_^2−^) [65]. Quaternary ammonium groups are highly stable and unaffected by pH change. Hence, they can captivate oxyanions of metals irrespective of the pH of the medium. Monomers, namely, (vinylbenzyl)trimethyl ammonium chloride, (3-acrylamidopropyl)trimethyl ammonium chloride and 2,3-epoxypropyltrimethylammonium chloride contains quaternary ammonium group as an active functional group in hydrogel preparation [66,67]. By ion exchange, hydrogels that consist of these monomers led to the exchange of the chloride (Cl¯) ions with oxyanions of metals.

### 6.2. Oxygen-Containing Functional Groups

#### 6.2.1. Hydroxyl Group

A hydroxyl (R-OH) group is composed of one oxygen atom bonded to one hydrogen atom. According to the International Union of Pure and Applied Chemistry (IUPAC), the word “hydroxyl” refers to a hydroxyl radical. A hydroxyl group can easily remove the proton to attract metal cations.

#### 6.2.2. Carboxyl Group

A carboxyl (R-COOH) group is composed of an electronegative oxygen atom that is double-bonded to the carbon atom and singly bonded to the –OH group. According to literature, the carboxyl group is found to be the most prominently used group to adsorb heavy metals onto the hydrogel surface. A carboxyl group gets ionized by giving an H^+^ ion from its R-OH group at alkaline pH, forming RCOO^−^ ion that readily attracts the divalent metal cations. To functionalize the carboxyl group onto the hydrogel surface, different methods used in post-treatment are surface grafting, etherification, and 2,2,6,6-tetramethylpiperidine-1-oxyl radical (TEMPO)-mediated oxidation, whichconverts a primary hydroxyl group into a carboxyl group [63].

### 6.3. Sulfur-Containing Groups

#### 6.3.1. Thiol Group

The thiol (R-SH) group plays an important role in functionalizing the hydrogel surface for heavy metal adsorption [68]. The thiol group acts as a Lewis base and interacts with the heavy metal (Lewis acid) by forming a coordinate bond [69]. From the literature, it is demonstrated that the thiol group shows strong bonding with mercury (Hg). The bonding can be well explained by Hard-Soft-Acid-Base (HSAB) theory, where mercury acts as a soft acid and prefers to bind with the thiol group (soft base) [70]. In an article by Kumar et al. the thiol group prefers to form a stable complex with highly polarizable soft heavy metals like mercury (Hg), gold (Au), and silver (Ag); and to a lesser extent with cadmium (Cd) and zinc (Zn), failing to form a coordinate bond with lighter metals like sodium (Na), calcium (Ca), and magnesium (Mg) [71].

#### 6.3.2. Sulfonic Acid Group

A sulfonic acid (R-SO_3_H) group is a sulfur-containing functional group that contains an electronegative sulfur atom that is double bonded to two oxygen atoms and single bonded to the –OH group. Sulfonic acid turns into sulfonate group (R-SO_3_^−^) on disassociation of hydrogen atom. Functionalizing a sulfonate group onto the surface of hydrogel makes the surface negatively charged, irrespective of the pH of the medium. A monomer 2-acrylamido-2-methyl propane sulfonic acid (AMPS) has been used to synthesize hydrogel via ^60^cobalt gamma-ray irradiation for the adsorption of Co^2+^, Mn^2+^, Cu^2+^,and Fe^3+^ [72].

### 6.4. Other Functional Groups

#### 6.4.1. Amidoxime Group

The general formula for the amidoxime group is (R-C(NH_2_)=N-OH). The amidoxime group forms stable complexes with many heavy metals such as Co^2+^, Cu^2+^, Ni^2+^, and Pb^2+^ but shows a strong affinity towards uranium. Therefore, hydrogel-based adsorbent material has been functionalized with the amidoxime group for the adsorption of uranium [73,74]. Guibal and coworkers synthesized amidoxime grafted chitosan magnetic hydrogel for sorption of uranium (U^6+^) and europium (Eu^3+^) [75]. Zeng et al. prepared amidoxime-modified hydrogel via graft copolymerization for adsorption of Cu^2+^. The maximum adsorption capacity of 40.7 mg/g at pH 5 was achieved over the contact time of 25 h [76].

#### 6.4.2. Phosphate-Containing Functional Group

Phosphate-containing functional groups, namely phosphine, phosphate, and phosphoramide are used to functionalize hydrogel surface. However, phosphate-based functional groups are more popular for functionalizing hydrogel for biomedical areas rather than metal adsorption. There are very few papers reported for metal adsorption. Liao et al. prepared phosphate functionalized graphene hydrogel for electrosorption of U^6+^. The maximum electrosorption capacity of 545.7 mg/g at 1.2 V and pH 5 was obtained [77].

#### 6.4.3. Chelating Group

The chelating agent such as aminopolycarboxylic acids (APCAs) helps in enhancing the adsorption affinity of the hydrogel by chelation [78]. Because there are many nitrogen- and oxygen-containing functional groups present in the aminopolycarboxylic acid structure. Especially, the nitrogen-containing functional group shows strong bonding interaction with divalent metal cations [79,80]. The four common aminopolycarboxylic acids for metal adsorption are ethylenediaminetetra acetic acid (EDTA), iminodiacetic acid (IDA), diethylenetriaminepentaacetic acid (DTPA), and nitrilotriacetic acid (NTA). Many biosorbents have been functionalized with EDTA because of their chemical stability, chelating ability, and low price [81]. IDA is a tridentate ligand forming a metal complex by chelation [82]. No studies have been reported for hydrogel surface modification by an NTA chelating agent. DTPA possesses five carboxylate groups, which show a high binding affinity for heavy metals after grafting on the hydrogel surface. For example, Huang et al. prepared DTPA-modified chitosan/alginate hydrogels for removal of Cu^2+^ from electroplating wastewater [83].

**Table 3:** Different hydrogel-based functionalized adsorbent materials for the removal of heavy metals.

Hydrogel	Active Functional Group	Heavy Metals Removed	References
Graphene oxide-chitosan-poly(acrylic acid) (GO-CS-AA) hydrogel nanocomposite	R-COOH	Pb^2+^	[84]
Hydrous ferric oxide-Poly(trans-aconitic acid/2-hydroxyethyl acrylate (HFO-P(TAA/HEA)) hydrogel	R-OH	Cu^2+^, Cd^2+^, Pb^2+^ and Ni^2+^	[85]
Chitosan-sodium lignosulfonate-acrylic acid (CS-SLS-AA) hydrogel	R-NH_2_	Co^2+^ and Cu^2+^	[86]
Poly(3-acrylamidopropyl) trimethyl ammonium chloride/ɤ-Fe_2_O_3_	R-N^+^(CH_3_)_3_	Cr^4+^	[87]
Sulfathiazole-based novel UV-curved hydrogel	R-SH	Hg^2+^, Cd^2+^ and Zn^2+^	[88]
Magnetic anionic hydrogel (nFeMAH)	R-SO_3_Na	Cu^2+^ and Ni^2+^	[64]
Poly(2-acrylamido-2-methyl-1-propane sulfonic acid) magnetic hydrogel	R-SO_3_H	Cd^2+^, Co^2+^, Fe^2+^, Pb^2+^,Cu^2+^, Cr^2+^ and Ni^2+^	[39]
Acrylamide/crotonic acid (AAm/CA) hydrogel	R-COOH, and R-CONH_2_	Hg^2+^	[89]
Glucan/chitosan hydrogel	R-OH and R-NH_2_	Co^2+^, Cu^2+^, Cd^2+^, Ni^2+^ and Pb^2+^	[90]
Malic acid enhanced chitosan hydrogel beads (mCHBs)	R-COOH and R-NH_2_	Cu^2+^	[91]
Carboxymethyl cellulose/polyacrylamide (CMC/PAM) composite hydrogel	R-OH, R-COOH and R-NH_2_	Cd^2+^, Pb^2+^ and Cu^2+^	[92]
Chitosan poly(acrylic acid) supermacroporous hydrogel	R-OH, R-COOH and R-NH_2_	Cu^2+^ and Pb^2+^	[93]
Lignosulfonate-modified graphene hydrogel	R-C=O, R-OH and R-COOH	Pb^2+^	[94]
Polyacrylonitrile-chitosan-graphene oxide (PCG) hydrogel composite	R-C(NH_2_)=N-OH	U^6+^	[73]

## 7. Synthesis of Hydrogel

A plethora of issues arising from the overuse of non-biodegradable materials and fossil resources have shifted researchers’ focus to renewable and environmentally friendly materials. In the present time, polymers are extensively used in different areas, namely agriculture, biomedical applications, wastewater treatment, and food packaging [95,96,97,98]. Similarly, for the removal of toxic heavy metals from wastewater by adsorption, polymeric hydrogels are the most promising adsorbent material due to their increased surface area, good solubility in organic solvents, improved functionality, low-priced, biodegradability, recyclability, enhanced adsorption capacity, and ease of fabrication. In addition, the excellent hydrophilic character makes these hydrogels suitable for wastewater treatment [35,99]. However, the effectiveness of the adsorbent material is highly dependent on the physicochemical properties of the adsorbent [100]. As a result, the first and most important step in developing an effective adsorption process is to synthesize a suitable hydrogel-based adsorbent material with high absorptivity of heavy metals present in wastewater.

The essential chemicals required for the synthesis of the hydrogel are a monomer, an initiator, and a cross-linker. Acryl amide (AAm), polyvinyl alcohol (PVA), polyvinyl pyrrolidone, acrylic acid (AA), 2-dimethylamino ethyl methacrylate (DMAEM), polyethylene glycol methyl ether methacrylate (PEGMEM), (3-Acrylamidopropyl) trimethylammonium chloride (APTMACI), N-isopropylacrylamide, 2-acrylamido-2-methyl-1-propan-sulfonic acid (AMPS), and 4-vinyl pyridine, 2-hydroxyethylmetacrylate are the examples of some monomers used in hydrogel synthesis [38,39,101,102,103,104,105,106]. Distinct monomers have different properties in terms of adsorption capacity, physical strength, and so on. In the synthesis of hydrogels, researchers were able to develop a solution to overcome the limitation of specific monomers. For example, to reduce the physical weakness of biopolymer chitosan, Sun et al. [107] and Liu et al. [108] used cellulose as the blending polymer in the synthesis of chitosan-based hydrogel for heavy metal adsorption.

Cross-linkers or cross-linking agents play a crucial role in the synthesis of polymeric hydrogels because they help to build up the polymeric three-dimensional network by stabilizing the binding sites amid the functional monomer and adsorption target molecule. Therefore, cross-linkers influence the polymers’ hydrophilic or hydrophobic properties, selectivity, mechanical stability, and morphology [109]. A cross-linker of organic or inorganic nature can be used in the synthesis process. Moreover, inorganic cross-linkers are mainly used to synthesize hydrogel adsorbents as organic cross-linkers that have certain disadvantages in terms of lower mechanical strength and thus cannot withstand stressed conditions; additionally, they also have a lower swelling capacity [41,110]. Furthermore, the characteristic properties of a hydrogel can differ depending on whether the cross-linking between the chains is covalent or non-covalent. Permanently cross-linked junctions exist in hydrogels that have been cross-linked with covalent bonds. Hydrogels cross-linked with non-covalent bonds (ionic interaction, hydrophobic interaction, or hydrogen bonding), on the other hand, have transient junctions [41,50].

For polymerization reaction, a cross-linker must have more than one active functional group to help linear polymer chains to join with other chains to form a stable three-dimensional structure. A low degree of cross-linking, in particular, corresponds to a small quantity of cross-linker, resulting in poor mechanical strength of polymeric material. As a result, the three-dimensional structure of hydrogel distorts during the application, and adsorption sites are disrupted, giving rise to a high number of non-specific perforations. When the degree of cross-linking is high, a densely packed three-dimensional mesh structure is generated, having excellent mechanical strength and an unexpectedly high mass transfer number. Resulting in the reduction of adsorption sites, as well as the degree of swelling of the hydrogel, causing heavy metals to barely penetrate the hydrogel surface. Therefore, it is optimal to maintain the quantity of the cross-linker in the specified range. Polymers having a cross-link ratio greater than 80% are generally used [109,111,112].

In the synthesis of hydrogels, an initiator is a chemical that helps to initiate the polymerization process. Table 4 summarizes various hydrogel-based adsorbents and the monomers, initiators, and cross-linkers used in their synthesis process.

### 7.1. Synthesis via the Chemical Route

Polymer chains in chemically cross-linked hydrogels are formed by covalent bonds. The subsequent sections describe various methods for the synthesis of the hydrogel by chemical modification.

#### 7.1.1. Chemical Route of Cross-Linking via Free Radical Polymerization

Free radical polymerization is one of the well-studied approaches for the synthesis of hydrogel in the presence of cross-linking agent N, N′ methylene bisacrylamide (MBA). The method involves three steps namely, initiation, polymeric chain propagation, and termination. In this process, the first step involves the generation of free radicals by using an initiator such as ammonium persulfate (APS), potassium persulfate (KPS), etc. in the vicinity of temperature, light, redox reaction, or ultraviolet or gamma radiation [121,122]. After that, in the second step, the free radical will react with the monomer to produce a radical monomer, which then reacts with the other monomers present in the solution to form polymeric chains. The cross-linker is added during the propagation of the polymeric chain, resulting in the formation of a three-dimensional structure of hydrogel. In the last step, the polymeric chain is terminated via disproportionation or combination reaction. The combination reaction connects two growing chains into one long polymeric chain. However, in the case of disproportionation reaction, a hydrogen atom is abstracted from the end of one growing chain and added to the other growing chain. As a result, a polymer with the unsaturated end group and a saturated end group is obtained. To speed up the process, an accelerating agent such as N,N,N′,N-tetramethylene diamine is added to the reaction mixture [123]. For instance, Shah et al. prepared superabsorbent polymer hydrogels containing acrylamide and acrylic acid as monomers via one-step free-radical polymerization. In this work, they aimed to remove multi-metals (Ni^2+^, Cd^2+^, Co^2+^, and Cu^2+^) from an aqueous medium [113].

#### 7.1.2. Chemical Route of Cross-Linking via High Energy Irradiation

The hydrogel synthesis via ultraviolet light radiation, electron beams, and ɤ-radiation is carried out at ambient or sub-ambient temperatures without the requirement of initiators, catalysts, or cross-linkers [124]. This synthetic route outperforms chemically initiated processes in regards to one-step hydrogel formation with no waste generation as a byproduct [125]. In this method, the density of cross-linking is estimated by duration and dose of irradiation. Polyethylene glycol (PEG), polyvinyl alcohol (PVA), alginate, chitosan, gelatin, hyaluronic acid (HA), carboxymethyl cellulose (CMC) are among the natural and synthetic polymers proposed for the hydrogel synthesis using this method [126,127]. This cross-linking method is similar to free radical polymerization in terms of three-step hydrogel formation: initiator, propagation of polymeric chain, and termination. When the mixture of the reaction solution is irradiated, a hydroxyl free radical is generated, resulting in the formation of a free-radical monomer. The hydrogel is synthesized when the network has reached the critical stage of gelling [128]. Maziad et al. prepared polyacrylic acid/polyvinyl alcohol based hydrogel to treat water decontamination via gamma radiation. They found that the hydrogel swelled 273%and had removal capacity of 150 mg/g, 155mg/g, and 193 mg/g for Ni^2+^, Co^2+^, and Cu^2+^ ions, respectively, at acidic pH 5 and after 24 h [129].

#### 7.1.3. Chemical Route of Cross-Linking via Grafting Reactions

In this method, the hydrophilic functional group like carboxyl (−COOH), sulfonic (−SO_3_H), amino (−NH_2_), and acylamino (−CONH_2_) are grafted on the surface of hydrogel [92,130]. Grafting the functional groups helps in improving the adsorption or desorption efficiency, as well as selectivity for specific heavy metals. As a result of this, there is an increase in surface polarity, hydrophilicity, and enhancement in the number of active sorption sites [131]. For example, Qi et al. prepared a new salecan polysaccharide-based hydrogel via graft copolymerization of sodium vinyl sulfonate and acrylamide onto the salecan for the effective decontamination of Pb^2+^ from wastewater [132].

#### 7.1.4. Chemical Route of Cross-Linking via Reaction of Functional Groups

The reaction involves the bond formation between the cross-linker and the functional moieties present in the polymer molecule. Hydrophilic groups such as amine (–NH_2_ in chitosan and proteins) and hydroxyl (–OH in cellulose and its derivatives) are bonded with cross-linking agents (such as glutaraldehyde) having an aldehyde functional group resulting in aldol product via covalent interaction. Hydrogel synthesis involving the polymers having hydroxyl groups needs certain specific conditions like methanol as a quencher, high temperature, and low pH. However, in the case of protein-based hydrogel no specific conditions are required [112,133,134]. Polymers with ester functional groups, on the other hand, undergo chemical cross-linking through the condensation process in the presence of a cross-linking agent, resulting in the formation of Schiff bases [130].

### 7.2. Synthesis via Physical Route

The physical route of cross-linking is highly favorable to synthesize non-toxic and environmentally friendly hydrogel as there is no requirement for chemical-based cross-linking agents [135]. In this process, polymer chains are held by weak interactions like hydrophobic interaction, ionic interaction, hydrogen bonding, Van der Waals forces, and π–π interaction [136]. From the literature, it is noted that polysaccharides like dextran, pullulan, carboxymethyl curdlan, and chitosan are used for the synthesis of hydrogels by this method [137]. 

#### 7.2.1. Synthesis via Freeze-Thaw

Crystallization via the freeze–thaw method is one of the physical processes used to synthesize hydrogel [138]. In this method, crystallization takes place by freezing low molecular solutes or bulk solvents, which enhances the polymer concentration by decreasing the chain gap and allowing the chains to align and join to create a three-dimensional structure [139]. In hydrogels, freeze–thaw cycles give rise to porous structures due to the space created by melting crystals during the thawing stages [140]. By varying the polymer concentration, the freezing temperature, freeze–thaw time duration, and the number of freezing and thawing cycles, the mechanical characteristics of the freeze–thawed hydrogel may be adjusted [139].

Hydrogels synthesized via the freeze–thaw method have greater elastic characteristics in comparison to those synthesized via chemical methods, attracting widespread interest across the world [141]. For instance, poly (vinyl alcohol) (PVA)/carboxy methyl cellulose (CMC) hydrogels are synthesized via the freeze–thaw method and used to absorb heavy metals such as Ni^2+^, Cu^2+^, Zn^2+^ and Ag^2+^ [139].

#### 7.2.2. Synthesis via Self-Assembling

Self-assembled hydrogels are prepared by monomeric units that spontaneously self-assemble by non-covalent interaction into supramolecular fibers [142]. When such fibers retain proper solvation in liquid (water), they efficiently entangle and immobilize solvent flow, resulting in a 3D mesh structure. The non-covalent interaction stabilizes hydrogel structures by making them softer than those generated by covalently cross-linked material [143]. These interactions provide self-assembled hydrogel with advantages such as tolerance to environmental perturbation and self-healing characteristics [144].

#### 7.2.3. Synthesis via Instantaneous Gelation

Another approach for synthesizing hydrogel quickly after a one-step procedure is instantaneous gelation [39,145]. For example, Zhou et al. synthesized novel chitosan-based magnetic hydrogel beads comprised of amine-functionalized magnetite nanoparticles, carboxylated cellulose nanofibrils, and polyvinyl alcohol incorporated chitosan for adsorption of Pb^2+^. The synthesized hydrogel beads exhibited an adsorption efficiency of 171.0 mg/g and could be regenerated in a weakly acidic solution with an adsorption efficacy of 90% after 4 cycles [146].

#### 7.2.4. Synthesis via Ionotropic Gelation

Hydrogel synthesis by ionotropic gelation allows the formation of microparticles and nanoparticles via electrostatic bonding among the two ionic species under suitable conditions, one of which must be a polymer [147]. For instance, sodium alginate(SA)/hydroxypropyl cellulose (HPC) hydrogel beads were synthesized with different ratios of 50:50, 75:25, and 100:0 for the removal of Pb^2+^. According to the results obtained, 75:25 showed better adsorption capacity in comparison to 50:50 and 100:0. After three hours of contact time, hydrogel beads showcased adsorption capacity and adsorption percentage of 47.72 mg/g and 95.45%, respectively [148].

#### 7.2.5. Synthesis via Inverse Emulsion Method

In the inverse emulsion method, the term “water-in-oil” describes the phenomenon in which water-soluble monomer is dispersed in the continuous phase oil (paraffin oil) by using an appropriate stabilizing agent, namely non-ionic surfactant Triton X-100, and after that the systems go through the phase inversion in a coagulation bath to release the monomer and precipitate out the porous film. This method has an advantage over other methods such as fine powdered product is obtained and by altering the reaction condition, the desired particle size can be achieved [149]. For example, a superabsorbent polymer-based hydrogel consisting of acrylic acid and carboxymethyl cellulose was synthesized by inverse emulsion polymerization method by using N, Nʹ methylene bisacrylamide (MBA) as a cross-linking agent and potassium persulfate (KPS) as an initiator. The maximum swelling capacity of 44.0 g/g in 0.9% *w*/*v* NaCl solution and 544.95 g/g in deionized water [150].

## 8. Characterization Techniques of Hydrogel

After the successful synthesis of hydrogel adsorbent, it becomes inevitable to investigate the physical, mechanical, structural, and morphological properties of the hydrogel formed. For this purpose, various characterization techniques such as Fourier transform infrared spectroscopy (FTIR), scanning electron microscopy (SEM), thermogravimetric analysis (TGA), zeta sizer, and energy-dispersive X-ray (EDX) are used to characterize the hydrogel (Table 5).

### 8.1. Functional Group Analysis

**Figure 6:**
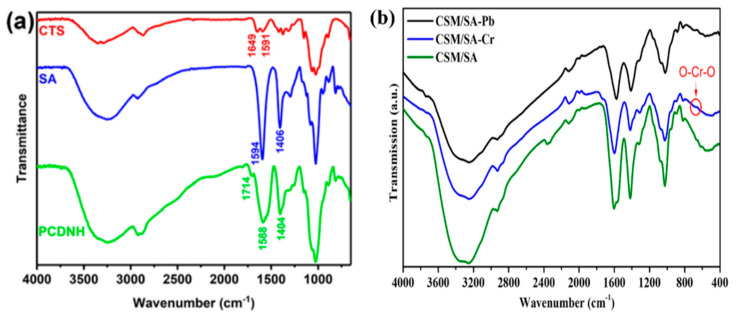
(**a**) FTIR spectra of chitosan (CTS), sodium alginate (SA), and PCDNH, (Reprinted from Ref. [151], Copyright (2022), with permission from Elsevier). (**b**) FTIR spectra of CSM/SA hybrid hydrogel beads loaded with Pb^2+^ and Cr^6+^ [152].

The surface functional groups such as hydroxyl, carboxyl, amide, amine, thiol, and amidoxime, etc. can be identified by using FTIR. Tang et al. synthesized chitosan/sodium alginate/calcium ion physically cross-linked double network hydrogel (PCDNH) for scavenging heavy metal ions. Analysis of FTIR spectra of hydrogels reveals that the peaks of chitosan at 1591 cm^−1^ and 1649 cm^−1^ (bending vibration of N-H and stretching vibrations for C=O of primary amine) disappear after the synthesis of PCDNH because the −NH_2_ group is converted to −NH_3_^+^. The symmetric and asymmetric stretching vibration peaks of −COO^−^ of sodium alginate at 1406 cm^−1^ and 1594 cm^−1^ shift to 1404 cm^−1^ and 1588 cm^−1^, indicating, the interaction between −COO^−^ with Ca^2+^ and −NH_3_^+^. A new peak was observed at 1714 cm^−1^ corresponding to the partial protonation of −COO^−^ after the formation of PCDNH (Figure 6a) [151]. Ablouh et al. investigated the adsorption of Cr^6+^ and Pb^2+^ via FTIR analysis for the preparation of Chitosan/Sodium alginate (CSM-SA) hybrid hydrogel beads. It was noticed that the peak of −NH_2_ or −OH at around 3250 cm^−1^ shifted to 3245 cm^−1^, indicating hydrogen bonding among the H atoms in −NH_2_ groups and the O atoms of oxyanions of Cr^6+^. In addition, there is a slight shift in the peak of COO from 1600 to 1590 cm^−1^, indicating the interaction between COO and Cr^6+^. These shifts correspond to electrostatic interaction between Cr^6+^ and NH_3_^+^, COO, and OH groups. A new peak observed at 682 cm^−1^ is due to the O-Cr-O band corresponding to Cr species. After the adsorption of Pb^2+^, the stretching vibration of OH and COO group shows a strong shift from 3250 to 3261 cm^−1^, and 1600 to 1569 cm^−1^, respectively. This shift is due to the coordination effect between Pb^2+^ and O atom, demonstrating ion-exchange among Ca^2+^ and Pb^2+^ on the surface of hydrogel (Figure 6b) [152].

### 8.2. Thermal Analysis

**Figure 7:**
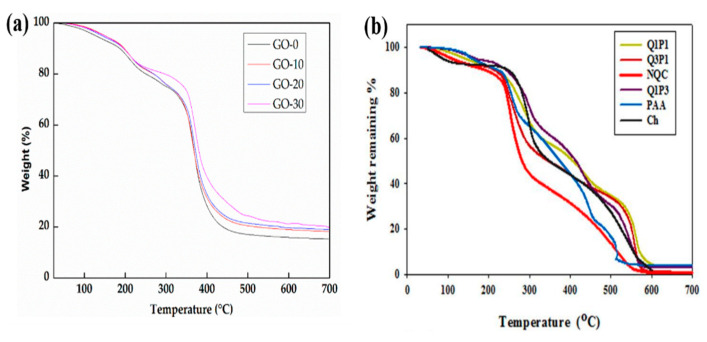
(**a**) TGA thermogram of the synthesized hydrogel with and without GO [153], and (**b**) Table 1. P1, Q1P3, and Q3P1. (Reprinted from Ref. [154], Copyright (2022), with permission from Elsevier).

Thermogravimetric analysis (TGA) is used to determine the thermal stability of a hydrogel. TGA can also be used to evaluate changes in a material’s physical and chemical properties as a function of increasing temperature. For example, Kong et al. studied the thermal stability of the Xylan-g-/p(acrylic acid-co-acrylamide)/graphene oxide (GO) hydrogel. Figure 7a represents the TGA thermogram of the hydrogel with and without GO. The weight loss of samples occurred in four phases when the temperature was raised from room temperature to 700 °C: 25–220 °C, 220–350 °C, 350–400 °C, and 400–700 °C. In the first step, weight loss was due to moisture loss and the decomposition of tiny molecules. The weight loss in the second step was because of the decomposition of long-chain compounds like polyacrylic acid, polyacrylamide, and xylan. The weight of hydrogels remained consistent in the last step, which was due to the carbonation of the hydrogels. Furthermore, the hydrogels with a higher GO loading had a high weight, indicating that GO has a positive effect on hydrogel thermal stability [153]. Mohamed et al. studied the thermal properties of a biodegradable N-quaternized chitosan (NQC)/poly (acrylic acid) (PAA) hydrogel by varying the NQC/PAA ratios to 3:1 (Q1P3), 1:1 (Q1P1), and 1:3 (Q1P3). The TGA thermogram revealed that the initial decomposition temperatures (IDT) of NQC, chitosan, PAA, Q3P1, Q1P1, and Q1P3 were observed at 214, 240, 229, 227,246, and 254 °C, respectively. Q1P3 hydrogel had the greatest IDT, indicating that it was the most thermally stable, owing to greater intermolecular hydrogen bonding between NQC and PAA chains. The thermal stability of hydrogels increased in the sequence: Q1P3 > Q1P1 > chitosan > Q3P1 > PAA > NQC (Figure 7b) [154].

### 8.3. SEM Analysis

**Figure 8:**
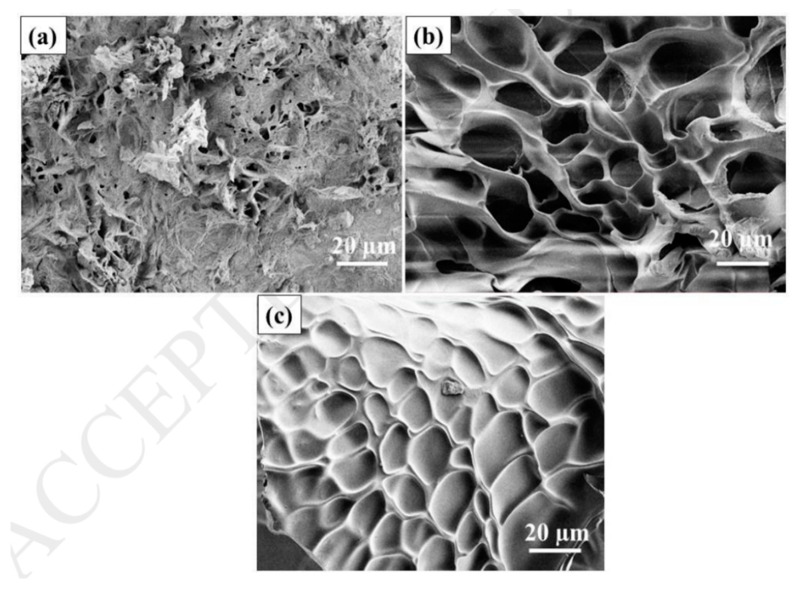
(**a**) CMC hydrogel, (**b**) CMC/PAM composite hydrogel, and (**c**) CMC/PAM composite hydrogel after the adsorption of Cu^2+^. (Reprinted from Ref. [92], Copyright (2022), with permission from Elsevier).

SEM is used to study the surface morphology, topography, and composition of the hydrogels. The porosity of hydrogel is a key factor attributed to its adsorption capacity. For instance, Godiya et al. synthesized bio-based carboxymethyl cellulose (CMC)/poly(acrylamide) (PAM) hydrogel for adsorption of heavy metals. SEM results demonstrated that CMC/PAM hydrogel has a sponge-like, three-dimensional, and highly mesoporous surface morphology (Figure 8b) that significantly differs from CMC hydrogel (Figure 8a). The CMC/PAM hydrogel has a pore size in the range of 5–15 µm in diameter. The pores developed in the hydrogel will permit guest molecules like water and heavy metals to move across the composite structure. The CMC/PAM composite hydrogel retained its structural robustness after the adsorption of Cu^2+^ (Figure 8c) [92].

**Figure 9:**
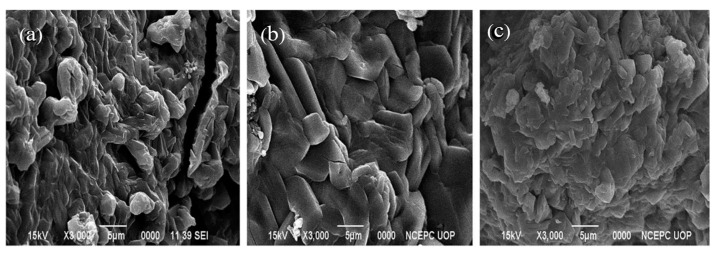
SEM micrograph of (**a**) anionic P(MAA), (**b**) neutral P(AAm), and (**c**) cationic P(APTMACI) hydrogels [155].

**Figure 10:**
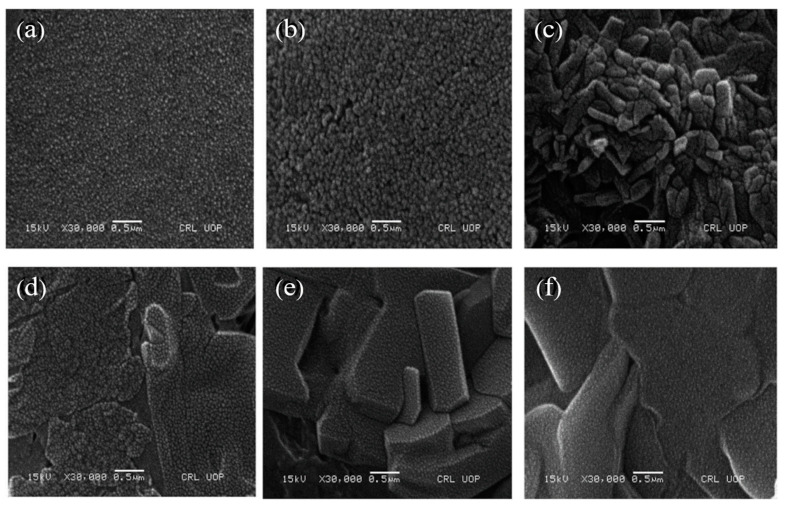
SEM micrographs of hybrid (**a**) P(MAA)-Cu, (**b**) P(MAA)-Ni, (**c**) P(APTMACI)-Cu, (**d**) P(APTMACI-Ni, (**e**) P(AAm)-Cu, and, (**f**) P(AAm)-Ni hydrogels [155].

Javed et al. synthesized anionic poly(methacrylic acid)(P(MAA)), neutral poly(acrylamide)(P(AAm)), and cationic poly(3-acrylamidopropyltrimethyl ammonium chloride)(P(APTMACI)) hydrogels and examined surface morphology by using SEM. SEM micrographs revealed that the surface P(MAA) was highly porous and rough compared to P(AAm) and P(APTMACI) (Figure 9a–c). The material with a rougher surface will generally have a higher adsorption capacity. As shown in Figure 10, SEM micrographs of hybrid hydrogels revealed that heavy metals nanoparticles were dispersed throughout the matrix without aggregation [155].

### 8.4. Zeta Potential Analysis

**Figure 11:**
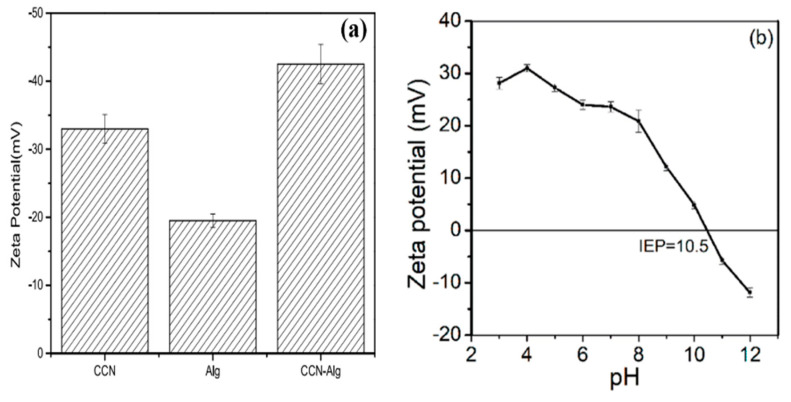
(**a**) Zeta potential (mV) of CCN, Alg, and CCN/Alg hydrogel beads. (Reprinted from Ref. [156], with permission from Elsevier), and (**b**) zeta potential of hydrogel beads varies as a function of pH, showing that negatively charged selenium absorbs at lower pH. (Reprinted from Ref. [158]. Copyright 2022 American Chemical Society).

Zeta potential is useful in determining the surface charge of hydrogel adsorbent. For example, Hu et al. synthesized carboxymethyl cellulose nanocrystals (CCN)/sodium alginate (Alg) hydrogel beads for scavenging Pb^2+^ and used a zeta-sizer to check the surface charge. Figure 11a depicts the zeta potentials of CCN, Alg, and prepared CCN-ALg were measured at pH 5.2. The zeta potential results revealed that all the three samples had negatively charged surfaces with stable dispersion at pH 5.2, with CCN-Alg being more so than the other two [156]. Bandara et al. studied the surface charge on chitosan/polyethylenimine/graphene oxide hydrogel beads for the abstraction of selenium from wastewater. Positive zeta potentials were noticed across a wide pH range, ranging from acidic pH to the isoelectronic point of 10.5, indicating ideal circumstances for electrostatic interaction with negatively charged species (Figure 11b) [157].

### 8.5. EDX Analysis

**Figure 12:**
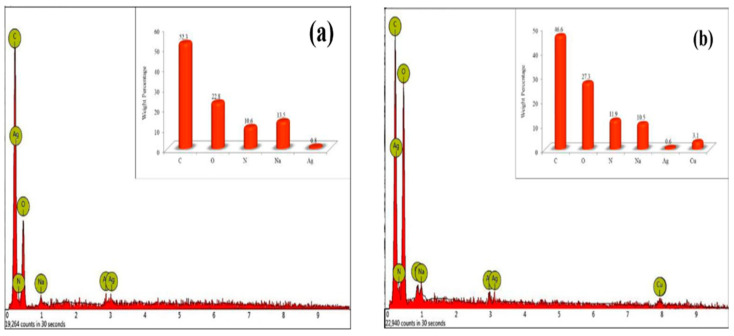
EDX spectra of NPGESNC-AcA (**a**) before Cu^2+^ adsorption, and (**b**) after Cu^2+^ adsorption (Reprinted from Ref. [121], Copyright (2022), with permission from Elsevier).

EDX characterization is used to determine the hydrogel’s elemental composition. Dil et al., for example, reported the fabrication of a novel porous gelatin-silver/poly (acrylic acid) (NPGESNC-AcA) nanocomposite hydrogel for Cu^2+^ removal. Figure 12 represents the element percentage of the synthesized NPGENC-AcA hydrogel before Cu^2+^ adsorption, which contains 52.3% carbon, 22.8% oxygen, 13.5% sodium, 10.6% nitrogen, 0.8% silver before adsorption of Cu^2+^. The results showed that silver was deposited in the nanocomposite hydrogel network, with no additional impurity elements detected in the spectrum (Figure 12a). EDX analysis for NPGENC-AcA after Cu^2+^ adsorption consists of 46.6% carbon, 27.3% oxygen, 11.9% nitrogen, sodium 10.5%, 0.6% silver, and 3.1% copper (Figure 12b) [121].

## 9. Adsorption Mechanism of Hydrogel

A thorough understanding of the adsorption mechanism and the removal process of various contaminants on different hydrogel-based adsorbents is essential for modifying hydrogels to enhance adsorption efficiency. The interactions such as electrostatic interaction, ion exchange, coordination interaction, and hydrophobic interaction take place depending on the surface functional moieties of hydrogels, provided reaction conditions such as temperature, pH, ligand, salt concentration, etc., and pollutant chemistry [159]. In literature, most hydrogel adsorbents are formed by the combination of interactions that take place simultaneously to form a 3D network. In the case of starch-based hydrogel, chemisorption and physisorption act simultaneously by acid-base interaction, H-bonding, ion exchange, or coordination interaction with heavy metal ions [160,161]. In chitin-based hydrogel, single or combination of multiple interactions occur depending on the operating condition and chemical composition [162]. In cyclodextrin-based hydrogel, complex formation occurs among the cyclodextrin and heavy metals involving host–guest interaction where hydrophobic bonding [163]. The various adsorption/desorption mechanism of heavy metals by hydrogel is discussed in Table 6.

### 9.1. Electrostatic Interaction

**Figure 13:**
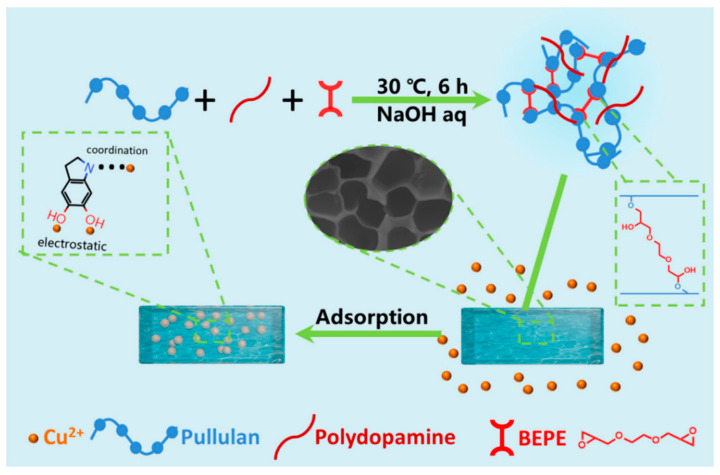
Schematic representation showcasing electrostatic and coordination interaction between pullulan/polydopamine hydrogel and heavy metals. (Reprinted from Ref. [158], Copyright (2022), with permission from Elsevier).

Electrostatic interaction occurs in the hydrogel with specific functional moieties in monomeric units having oppositely charged ions such as cation–anion interaction concerning heavy metals that need to be adsorbed or desorbed [159]. Furthermore, the pH of the solution has a significant impact on the generation of charged ions on the adsorbent (hydrogel) surface [164]. The pH of the solution is represented by pH_PZC_ when there are no charged ions on the surface of the adsorbent [165,166]. When the pH > pH_PZC_, the surface functional moieties like −OH, −COOH, and −H_3_PO_4_ lose the proton due to the higher concentration of OH^−^ ions in the solution that forms anions like −O^−^, −COO^−^, −PO_4_^3−^ etc. on the surface of the adsorbent. However, at pH < pH_PZC_, the surface of the adsorbent is positively charged due to an increase in the concentration of H^+^ ions, which causes protonation of functional moieties such as −SH, −NH_2_, etc [167]. According to the studies reported, electrostatic interactions are the dominant adsorption force for heavy metals abstraction in various hydrogels. Yu and co-workers synthesized sodium alginate(SA)/carboxylated nanocrystals cellulose hydrogel beads for the abstraction of Pb^2+^. The findings in this study reveal that the adsorption mechanism that took place was complexation among −COO and −OH functional moieties and heavy metal (Pb^2+^) by sharing a pair of electrons. Thereafter, the electrostatic interaction was found to occur between negatively charged hydrogel beads and positively charged Pb^2+^ ions [156]. Tang et al. synthesized physically cross-linked double network hydrogel (PCDNH) containing chitosan, calcium ion, and sodium alginate. In this study, they reported that chitosan’s cationic NH_3_^+^ group reacts with sodium alginate’s anionic −COO^−^ group to construct physically cross-linked hydrogel via electrostatic interaction. In addition, the adsorption of heavy metals (Pb^2+^ and Cd^2+^) on the hydrogel surface was due to the electrostatic interaction with PCDNH’s oxygen atom, whereas the adsorption of Cu^2+^ was primarily due to coordination interaction with PCDNH’s nitrogen atom, besides electrostatic interaction [151]. Zeng et al. prepared pullulan/polydopamine hydrogel for effective elimination of heavy metals (Co^2+^, Cu^2+^, and Ni^2+^). In this research work, hydrogels were prepared by chemically cross-linking pullulan with 1,2-bis (2,3-epoxypropoxy) ethane. Polydopamine was added to the mixture to form a novel hydrogel adsorbent. Polydopamine’s nitrogen atom and catechol group have a high affinity to react with positively charged metal-ion via electrostatic and coordination interaction (Figure 13) [158].

### 9.2. Ion-Exchange

**Figure 14:**
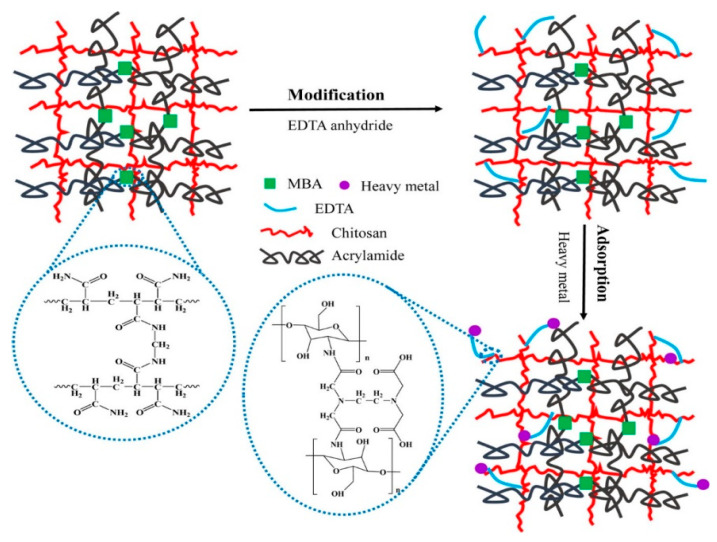
The proposed mechanism between chitosan/polyacrylamide hydrogel and heavy metal ions. (Reprinted from Ref. [115]. Copyright 2022 American Chemical Society).

Ion exchange refers to a chemical process whereby the swapping of ions takes place between an insoluble adsorbent (hydrogel) and a liquid phase (wastewater). The unwanted anions or cations dissolved in the wastewater are replaced or removed by the ions of a similar charge present on the hydrogel surface. To maintain the neutrality of the system the number of ions adsorbed by the hydrogel adsorbent must be equal to the number of ions liberated [168]. Ion exchange provides an efficient and convenient route becauser it can distillate and separate distinct contaminants from wastewater [169]. It reduces the degree of harmful load by converting heavy metals waste into a form that can be reused and recycled, leaving behind less hazardous materials in the solution, or by reducing the hydraulic flow of the stream containing toxic heavy metals, allowing for the final release [167]. Ion-exchange mechanisms, like electrostatic interaction, are highly dependent on the pH of the solution. Due to a rise in the concentration of H^+^ ions at pH<pH_PZC,_ the functional moieties in hydrogel adsorbent become positively charged, leading to cation exchange. However, at pH > pH_PZC_ the functional moieties are negatively charged due to excessive concentration of OH^-^ ions, leading to anion exchange [170]. Saber-Samandari et al. synthesized Cellulose-Graft-Polyacrylamide/Hydroxyapatite hydrogel composite for the removal of Cu^2+^ ions. In this work, he observed that Cu^2+^ ions got exchanged with the cations in the hydrogel composite and are attached to the surface of hydroxyapatite by an ion-exchange mechanism [171]. For the treatment of heavy metals from oily wastewater, Xiong et al. prepared a self-cleaning cellulose functionalized titanate microsphere hydrogel via a sol-gel method. The prepared hydrogel microspheres have the combined properties of cellulose and titanate nanotubes that exhibit a high capacity to maintain oily wastewater. At first, Cu^2+^ got absorbed on the inner surface of cellulose titanate hydrogel by electrostatic interaction. After that, Cu^2+^ was captured in the layer of titanate nanotubes exhibiting remarkable characteristics for heavy metals under the influence the chemical and physical adsorption [172]. Ma et al. prepared ethylenediaminetetraacetic acid (EDTA) functionalized double network hydrogel for efficient elimination of heavy metals (Cd^2+^, Pb^2+^, and Cu^2+^) from industrial eluents. In this work, a two-step process was conducted in which first polyacrylamide was cross-linked with N, N′ methylene bisacrylamide (MBA), and then EDTA was cross-linked with chitosan to form a double network hydrogel. The hydrogel showed a maximum sorption capacity of 138.41 mg/g, 99.44 mg/g, and 86.00 mg/g for Pb^2+^, Cu^2+^, and Cd^2+^ respectively based on the ion exchange mechanism between carboxylate groups and heavy metal ions (Figure 14) [115].

### 9.3. Hydrophobic Interaction

**Figure 15:**
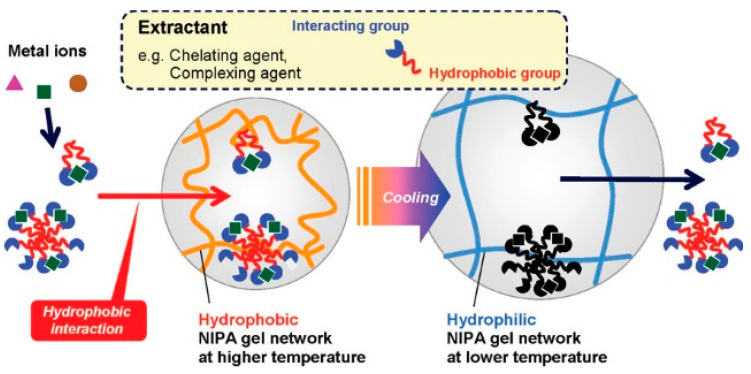
The proposed mechanism of heavy metal complexed with extractant onto N-isopropyl acrylamide hydrogel. (Reprinted with permission from Ref. [174]. Copyright 2022 American Chemical Society).

The interaction taking place between water molecules and hydrophobes (non-polar molecules containing long carbon chains that do not react with water molecules because of weak Van-der-Waals forces) is termed hydrophobic interaction [173]. Therefore, a low water-soluble molecule is more likely to be attracted by hydrophobes. For example, Tokuyama et al. prepared superabsorbent hydrogel containing N-isopropyl acrylamide (NIPA) as a thermo-responsive polymer for heavy metals extraction. At first, an aqueous solution of metal ions is complexed with an extractant that has a hydrophobic group and an interacting group. After that, above lower critical solution temperature the complex formed between metal and extractant gets absorbed into the hydrogel via hydrophobic interaction. Finally, after cooling below the low critical solution temperature metal-extractant complexes are extracted from the hydrogel. In this study, Cu^2+^ is used as a model heavy metal ion [174]. The mechanism for the same is depicted below in Figure 15.

### 9.4. Coordination Interaction

**Figure 16:**
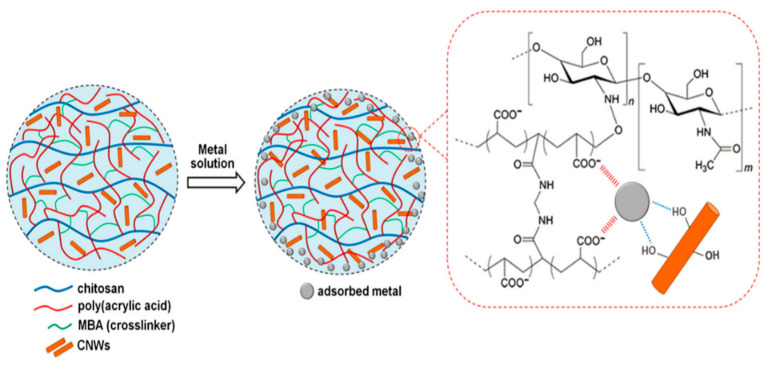
The coordination interaction between chitosan-g-poly (acrylic acid)/cellulose nanowhiskers hydrogel beads and adsorbed metal. (Reprinted by permission from Ref. [176]. Copyright (2022), Springer).

Coordination interaction also known as chelation interaction refers to the formation of covalent bond where a single-atom shares both the electrons. In this interaction, cation (heavy metals) binds with the group containing lone pair electrons, resulting in cation adsorption on the adsorbate surface [167]. Zhaung et al. prepared double network alginate/graphene nanocomposite hydrogel beads for effective extraction of Cu^2+^ and dichromate (Cr_2_O_7_^2−^). He observed that –COOH functional moieties in both graphene and alginate show a high affinity for Cu^2+^ and Cr_2_O_7_^2−^ via coordination and complexation. On the contrary, ion exchange takes place between Ca^2+^ ions in alginate and Cu^2+^ in the aqueous solution [175]. Rodrigues et al. prepared chitosan-g-poly (acrylic acid)/cellulose nanowhiskers (CNWs) composite hydrogel beads by using N, N′ methylene bisacrylamide as a cross-linker for the adsorption of Cu^2+^ and Pb^2+^ from water. FTIR analysis revealed that functional moieties i.e., hydroxyl groups and carboxyl groups act as coordination sites for heavy metal adsorption [176]. The schematic representation depicting the coordination between hydrogel adsorbent and heavy metals is demonstrated in Figure 16.

**Table 6:** Proposed synthesis and removal mechanism of various hydrogel-based adsorbents.

Hydrogel Type	Synthesis Method	Mechanism	Heavy Metals Removed	References
Carboxymethyl cellulose-graft-poly(acrylic acid)/monmorillonite hydrogel composite	Graft polymerization	Ion exchange and coordination interaction	Zn^2+^, Pb^2+^	[177]
Silk sericin/Lignin hydrogel beads	Graft polymerization	Ion exchange or electrostatic interaction	Cr^6+^	[178]
Chitosan/multiwall carbon nanotube/poly(acrylic acid)/poly(4-aminodiphenyl amine) functional gel	Free radical polymerization and cross-linking reaction	Complexation interaction	Cr^6+^	[60]
Sugar cane bagasse cellulose and gelatin-based hydrogel composite	Cross-linking	Coordination and electrostatic interaction	Cu^2+^	[179]
Carboxy methyl cellulose hydrogel	ɤ-raddiation	Coordination interaction	Cu^2+^	[180]
Chitin/cellulose composite hydrogel	Freeze-thaw method	Electrostatic and coordination interaction	Hg^2+^, Cu^2+^, Pb^2+^	[181]
Carboxy methyl cellulose hydrogel beads	Inverse suspension method	Coordination interaction	Cu^2+^, Ni^2+^, Pb^2+^	[182]
Hydrogel-biochar composite	Free radical polymerization and cross-linking reactions	Chemisorption	As	[101]
Pullulan/polydopamine hydrogels	Chemical cross-linking	Electrostatic and coordination interaction	Cu^2+^	[158]
Jute/poly(acrylic acid) hydrogel	Free radical polymerization	Electrostatic interaction	Cd^2+^, Pb^2+^	[183]
Carboxylated chitosan/carboxylated nanocellulose hydrogel beads	Cross-linking	Electrostatic and coordination interaction	Pb^2+^	[184]

## 10. Recovery, Regeneration, and Reusability of Hydrogel

One of the paramount characteristics of hydrogel other than high adsorption efficiency is their regeneration capacity by desorbing the absorbed heavy metals which further allows it to be reused. The ability to regenerate and reuse an adsorbent material is also an important factor for the practical assessment of its application. Many different ways have been studied by researchers for the effective desorption of heavy metals from the three-dimensional mesh structure of hydrogel after every removal cycle. Changes in the magnetic field, electric field, temperature, pH, etc. will lead to the desorption of heavy metals [185]. The influence of pH on heavy metal desorption from a magnetized cellulose-chitosan hydrogel was reported by Liu et al. [108]. At very low pH values of 1.0–2.0 desorption efficiency of 83–86% was achieved. This represents the merits of using pH-dependent hydrogel for the adsorption of heavy metals such as arsenic and chromium during the elimination process and desorption for the recovery of hydrogels. Moreover, adjusting the required pH is a drawback [38]. According to the literature, studies reported on the recovery of hydrogel adsorbents have used strong as well as weak acids as eluents (HCl, HNO_3_, CH_3_COOH, H_2_SO_4_, etc.) [37]. Furthermore, the type of acid utilized in the desorption process also has a considerable impact on the durability and desorption capacity of hydrogel [24]. Mohammadi et al. synthesized a chelator-mimetic multi-functionalized hydrogel with a high metal adsorption efficiency (cadmium, lead, and arsenic) and great reusability. By employing a low concentration of hydrochloric acid, the heavy metals absorbed in the hydrogel network were eluted and the hydrogel was regenerated for reuse. After five adsorption/desorption reuse cycles, a removal ratio greater than 60% was obtained [185]. By applying a similar approach, Pourjavadi et al. developed a novel hydrogel containing chitosan, acrylic acid, and an amine-functionalized nano-silica. In this work, 1M hydrochloric acid solution was employed for recovering hydrogel loaded with Pb^2+^. The hydrogel was then regenerated by filtering and washing with deionized water before being utilized for the next adsorption cycle. After three consecutive cycles, the efficiency of regenerated hydrogel remained around 685–715 mg/g [186].

**Figure 17:**
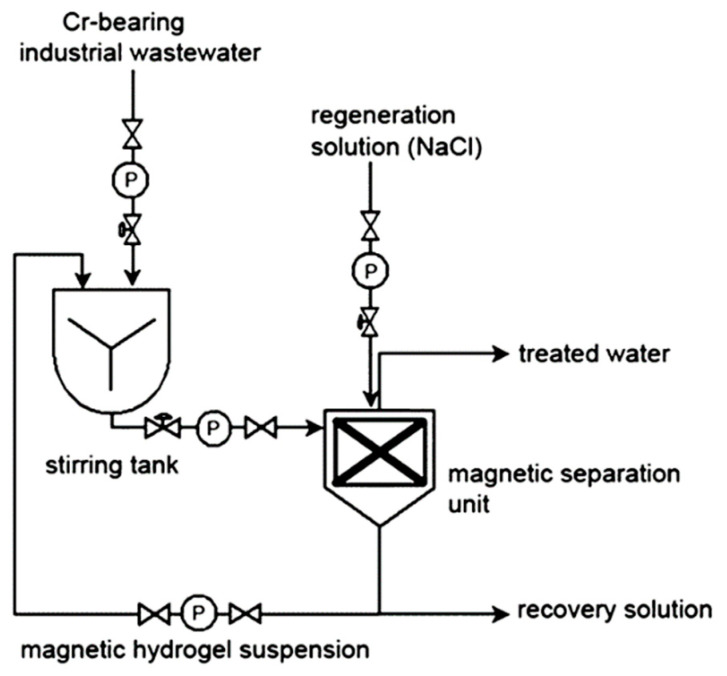
Schematic representation of a wastewater treatment experiment with a magnetic separation unit. (Reprinted with permission from Ref. [188]. Copyright 2022 Americal Chemical Society).

Magnetic hydrogels are one of the most used adsorbent materials for the effective elimination of heavy metals from flowing streams. During the recovery process, the eluent acidity needs to be managed since an excess of acid can damage the magnetic adsorbent. Eluents with high concentrations can damage the binding sites on hydrogels, resulting in lowered adsorption efficiency after numerous sorption cycles [187]. Tang et al. synthesized a magnetic hydrogel with high adsorption efficiency of 200 mg/g for Cr^6+^ adsorption. The hydrogel possesses an advantage of easy recovery by regenerating in sodium chloride solution (NaCl) [188]. The applicability of any magnetic hydrogel adsorbent in contaminated water is determined by two important factors: an increase in the concentration of heavy metals in that solution and a lower quantity of recovery solution. Tang’s research summarized both the factors in Figure 17. In brief, the treated contaminated water is collected and separated from hydrogel in a magnetic separation unit; NaCl at different concentration is injected for the regeneration of hydrogel, and the leftover solution were collected by separating the magnetic hydrogel. A series of regeneration tests were carried out by step-wise addition of sodium chloride solution. The recovery solution was then collected and further processed by the addition of NaCl solution to it. The results obtained suggested recovery efficiency was maintained for 20 sorption cycles, resulting in the Cr^6+^ removal capacity of 97–98%. According to the results achieved, the Cr content in the recovery solution reached 500–600 mg/L corresponding to wastewater:recovery volume ratio of 40:1 [188].

Reusability of hydrogel is one of the most important characteristics for wide-range applications, although it is a challenge for conventional hydrogel adsorbents as they possess poor mechanical strength after swelling in aqueous media. Therefore, increasing mechanical strength plays a crucial role in maintaining the desired adsorption efficiency of the heavy metals-loaded hydrogel. Liu et al. reported that 95% Fe, Pb, and Cu were removed from an aqueous medium through 7 adsorption cycles and hydrogel still can lead to heavy metals removal [108]. Therefore, it proves that hydrogel can be reused many times and lowers the cost of production for heavy metals elimination from an aqueous solution. Tang et al. reported the reusability and regeneration of hydrogel in a column experiment for effective elimination of Cr^6+^ by a cationic hydrogel. After 6 sorption cycles adsorption efficiency remained constant (27 mg g^−1^, 90%) and the desorption capacity was 93 percent on average for every cycle [189]. In conclusion, it can be said that low operational and production-cost along with easy separation capabilities and reusability make hydrogel a choice of adsorbent for heavy metal removal from wastewater.

## 11. Conclusions

Water pollution is one of the serious global problems caused by increasing industrialization and urbanization. In particular, heavy metals discharged into flowing streams have detrimental effects on human health and the natural ecological system. Thus, it is necessary to treat the wastewater containing toxic heavy metals and then discharge it. The adsorption process including various types of adsorbent material is regarded as an efficient, cost-effective, and environment-friendly approach to the treatment of heavy metals. However, the majority of adsorbent materials used for wastewater treatment are non-biodegradable, synthetic, and require post-treatment after use, which prompts researchers’ interest in developing biodegradable, easy to modify, and biocompatible adsorbent materials. Hydrogels as potential adsorbent materials represent the best choice. The present review summarizes the literature concerning hydrogels in the past 25 years, and describes the classification, properties, synthesis, mechanism and recovery, regeneration, and reuse of hydrogel-based material for the elimination of heavy metals.

Although hydrogels have been extensively studied, there are still a few areas that require further investigation.

- Currently, the hydrogel-based adsorbent materials used for heavy metal removal are limited to lab scale. Therefore, further research is required to scale up for a large-scale application.
- The present research is confined to removing a single type of heavy metal. More research should be undertaken targeting multiple heavy metals.
- The research should focus on the ability of the hydrogel to regenerate (for example, the adsorption efficiency of hydrogel drops after five sorption cycles).
- To broaden the spectrum of hydrogels application for separation of rare earth metals.
- To develop high mechanical strength tailored hydrogel (for example hydrogel membranes) that are easier to separate from the liquid phase for wastewater treatment.

## Figures and Tables

**Table 4:** Different hydrogel adsorbents and associated monomers, cross-linker, and initiators in the synthesis process.

Hydrogel	Monomer	Cross-Linker	Initiator/Accelerator	References
Poly(2-acrylamido-2-methyl-1-propansulfonic acid-co- vinylimidazole) hydrogel	2-acrylamido-2-methyl-1-propansulfonicacid (AMPS), N- vinyl imidazole	N,N′ methylenebisacrylamide (MBA)	2,2′-azobis(2-methyl propionamide) (MPA) dihydrochloride	[37]
Cationic hydrogel	(3-acrylamidopropyl) trimethylammonium chloride (APTMCI)	N,N′ methylenebisacrylamide (MBA)	Ammoniumpersulfate (APS)/N,N,N′,N′-tetramethylenediamine (TEMED)	[38]
Hydrogel biochar composite	Acrylamide (AAm)	N,N′ methylenebisacrylamide (MBA)	Ammonium persulfate (APS)	[101]
Fe_2_O_3_ nanoparticles functionalized polyvinyl alcohol/chitosan magnetic composite hydrogel	Polyvinyl alcohol (PVA)	Glutaraldehyde vapor	Glacial acetic acid	[102]
Methacrylate-based hydrogel	Polyethylene glycol methyl ether methacrylate (PEGMEM), 2-dimethylamino ethyl methacrylate	N,N′ methylenebisacrylamide (MBA)	Ammonium persulfate (APS)	[104]
(p-4-VP-co-HEMA) composite hydrogel	4-vinyl pyridine (4-VP), 2- hydroxyethylmetacrylate (HEMA)	N,N′ methylenebisacrylamide (MBA)	Ammonium persulfate (APS), N,N,N′,N′-tetramethylenediamine (TEMED)	[106]
Chitosan-cellulose hydrogel	Chitosan	Cellulose	-	[107]
Superabsorbent polymer hydrogels	Acrylic acid (AA), acrylamide (AAm)	N,N′ methylenebisacrylamide (MBA)	Ammoniumpersulfate (APS)	[113]
Poly(N-hydroxymethylacrylamide) hydrogel	N-hydroxymethylacrylamide	Polyethylene glycol (400) diacrylate	Ammonium persulfate (APS)/N,N,N′,N′-tetramethylenediamine (TEMED)	[114]
EDTA Functionalized Chitosan/Polyacrylamide double network hydrogel	Chitosan, acrylamide	N,N′ methylenebisacrylamide (MBA)	Potassium persulfate (KPS)	[115]
N-vinyl-2-pyrrolidone/Itaconic acid hydrogel	Itaconic acid (IA), N-vinyl-2-pyrrolidone	N,N′ methylenebisacrylamide (MBA)	Ammoniumpersulfate (APS/N,N,N′,N′- tetramethylenediamine (TEMED)	[116]
Polyampholyte hydrogel	Methyl methacrylate (MMA), acrylic acid (AA)	N,Nʹ methylenebisacrylamide (MBA)	Ammonium persulfate (APS)/N,N,N′,N′- tetramethylenediamine (TEMED)	[117]
Poly(acrylic acid) hydrogel adsorbent	Acrylic acid (AA)	Calcium hydroxide (Ca(OH)_2_) nano-spherulites (CNS)	Ammonium persulfate (APS)/N,N,N′,N′-tetramethylenediamine (TEMED)	[118]
Magnetic chitosan hydrogel beads	Chitosan	Glutaraldehyde	-	[119]
Hydrogel-based on novel cross-linker	Chitosan, acrylic acid, glucose	Allyl pentaerythritol(AP)^15^/allyl mannitol (AP)^14^/allyl sorbitol	Potassium persulfate (KPS)	[120]

Hydrogels are synthesized via two routes: Chemical and physical.

**Table 5:** Characterization techniques used for hydrogel adsorbent and information obtained from the characterization tools.

Characterization Techniques	Characteristics
Fourier Transform Infrared Spectroscopy (FTIR)	Functional group
Field Emission-Scanning Electron Microscopy (FE-SEM)	Surface morphology
Thermo Gravimetric Analysis (TGA)	Thermal stability
Zeta Sizer	Surface charge
Energy Dispersive X-ray (EDX)	Elemental composition

## Data Availability

Not applicable.
